# Cranial Morphological Patterns Based on Cranial Index and Petrous Ridge–Midline Angle in Koreans: Implications for Clinical and Forensic Applications

**DOI:** 10.3390/diagnostics15212802

**Published:** 2025-11-05

**Authors:** Digud Kim, Jeonghyun Park, Jaeho Cho, Yu-Jin Choi, Hyung-Wook Kwon, Yunil Choe, Sookyoung Lee, Kwang-Rak Park

**Affiliations:** 1 Department of Anatomy & Cell Biology, College of Medicine, Kangwon National University, Chuncheon-si 24341, Gangwon-do, Republic of Korea; oe5235@naver.com (D.K.); jhpark@kangwon.ac.kr (J.P.); police5565@hanmail.net (Y.-J.C.); kwenhw@naver.com (H.-W.K.); loloo4ve@naver.com (Y.C.); 2 Department of Orthopedic Surgery, Chuncheon Sacred Heart Hospital, College of Medicine, Hallym University, 77, Sakju-ro, Chuncheon-si 24253, Gangwon-do, Republic of Korea; hohotoy@nate.com; 3 Division of Forensic Medical Examination, National Forensic Service, 10, Ipchun-ro, Wonju-si 26460, Gangwon-do, Republic of Korea; heart@korea.kr; 4 Department of Anatomy, College of Korean Medicine, Sangji University, Wonju-si 26339, Gangwon-do, Republic of Korea

**Keywords:** cranial morphology classification, cranial index, petrous ridge–midline angle, post-mortem computed tomography, Koreans

## Abstract

**Background:** The human skull has a very complex and diverse structure, and morphological characteristics vary by population. The purpose of this study is to comprehensively analyze the cranial morphology using postmortem computed tomography (PMCT), and to identify anatomical characteristics through a multifaceted approach in Koreans. **Methods:** 358 PMCT cross-sectional images (179 males, 179 females) provided by the National Forensic Service were analyzed, and the average age was 55.1 ± 18.9 years. The maximum cranial length was divided by the maximum cranial width and multiplied by 100 to calculate the cranial index (CI). Petrous ridge–midline angle (PMA) was measured as the angle between the petrous ridge and the midline. **Results:** In both the classification of skull shape using CI and PMA, the brachycranic type showed the highest frequency (*p* < 0.001). Classified by CI, there were no significant differences in frequency by sex (*p* = 0.533), or age (*p* = 0.110). However, classified by PMA, the frequency of the brachycranic type in men was significantly higher than in women (*p* = 0.022), and there was a significant difference in the frequency of cranial morphology by age (*p* < 0.001). **Conclusions:** The results of cranial morphology classification targeting Koreans are expected to provide useful basic data for clinical and forensic use.

## 1. Introduction

Cranial morphology is one of the important factors that represents racial characteristics in terms of physical anthropology. It is known to be less influenced by environmental factors and more influenced by genetic factors [1,2]. Information on cranial morphology is a source of identification for unidentified human bodies. It has important uses in forensic, anthropological, archaeological and anatomical research. It is closely related to clinical diagnosis and treatment in the evaluation of growth, development, and clinical disorders. Therefore, the application of a detailed and precise classification of cranial morphology is necessary [2,3].

The methods for measuring cranial morphology can be divided into direct and indirect methods. The indirect method is a method of investigating the morphological variation or frequency of occurrence of specific parts of the sutures and foramens of the skull. The direct method is to measure distances or angles between various points on the skull [3,4]. A representative direct method is the cranial index (CI), which is calculated using the maximum cranial length (MCL) and maximum cranial width (MCW). It has been used to classify cranial morphology by population or to estimate the population, age, gender, height, and facial shape of unidentified individuals according to the occurrence of a mass disaster or the passage of time of death [2,5,6].

The previous studies using the CI have been used to classify cranial morphology in various populations. However, there are some lacking parts in accurately describing the very complicated types of skulls, so the application of more precise and detailed methods is required [1,4,7,8]. Eskandary et al. [9] refined the classification of cranial morphology by introducing a method that measures the angle between the petrous ridge and the midline, known as the petrous–midline angle (PMA), in addition to the conventional cranial index (CI) method. They argued that cranial morphology varies across populations and that the same skull can be categorized into different and more refined types depending on the applied method, such as CI or PMA. Therefore, in this study, the PMA method, which measures the angle between the petrous ridge and the midline, was additionally utilized to classify cranial morphology. It was judged that applying both the CI and PMA methods would allow for a more advanced and detailed classification of cranial morphology.

The purpose of this study is to comprehensively analyze PMCT images using two craniometric methods, and to identify anatomical characteristics of Koreans through a multifaceted approach.

## 2. Materials and Methods

### 2.1. Study Design

**Figure 1:**
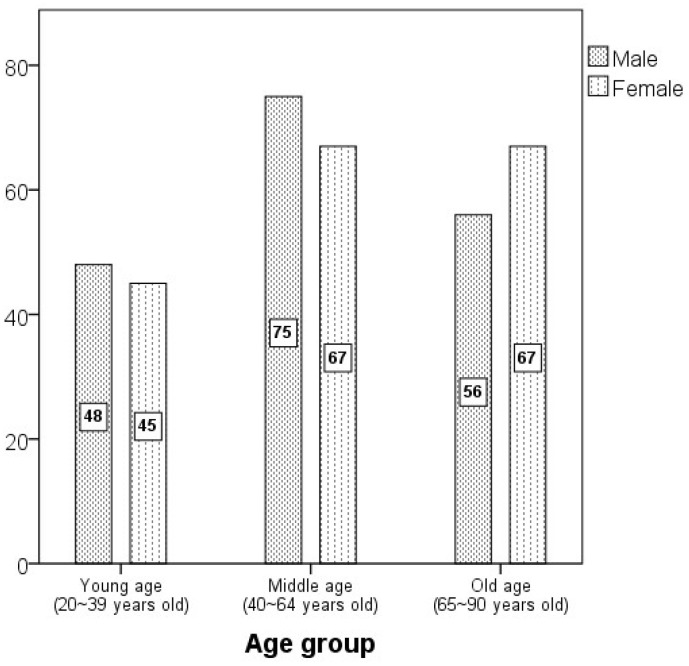
The distribution graph by sex and age group (*N* = 358).

In this study, 358 PMCT images (179 males, 179 females) examined at the National Forensic Service (NFS) were analyzed from January 2020 to December 2022. The study was conducted with the approval of the Institutional Review Board (IRB) of the NFS (IRB No. 906-250319-HR-004-05). It was designed as a retrospective analysis using postmortem computed tomography (PMCT) data. As all subjects were deceased, obtaining written informed consent was not possible. In accordance with the Bioethics and Safety Act and related Korean regulations, the requirement for informed consent was officially waived by the Institutional Review Board after confirming that the data were fully anonymized, that there was no reasonable basis to assume refusal of consent owing to the subjects’ death, and that the study posed minimal or no risk to the individuals involved. The average age of the deceased at the time of imaging was 55.1 ± 18.9 years (21–89 years). Based on age, the subjects were classified into three groups: young age group (20–39 years), middle age group (40–64 years), and old age group (65–90 years) (Figure 1). The images used in the study were selected based on the following criteria: no fractures, deformities, injuries, diseases, or surgical abnormalities in the cranial region; and the direct or indirect cause of death was unrelated to the cranial region. Additionally, the determination of proper imaging posture was based on the simultaneous visualization of both infraorbital margins, supraorbital margins, and both external auditory canals in cross-sectional images captured in a neutral position.

### 2.2. PMCT Imaging and Data Acquisition

In this study, PMCT scans were acquired using a PMCT system (Aquilion PRIME, CANON Medical Systems, Otawara, Japan) at the NFS under the following conditions: 120 kVp tube voltage, pitch factor 0.637, and slice thickness 1.0 mm. Images were analyzed under bone settings (window width 1500, window level 500) using a DICOM viewer (version 0.1.5 Beta, MicroDicom, MicroDicom Ltd., Sofia, Bulgaria).

### 2.3. Cranial Measurement and Morphological Classification

**Figure 2:**
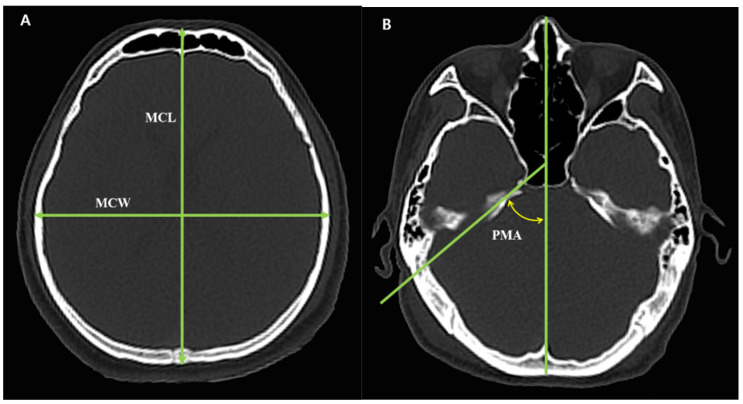
Two methods for cranial morphology measurement. (**A**) CI measurement method and section level; (**B**) PMA measurement method and section level. MCL, Maximum cranial length; MCW, maximum cranial width; PMA, Petrous ridge–midline angle; Cranial index (CI) = (MCW/MCL) × 100.

Using the DICOM viewer, the MCL was measured at the longest point of the anterior–posterior length of the skull, and the MCW was measured at the longest point of the left-right width of the skull. The CI was calculated by dividing the MCW by the MCL and multiplying by 100. The PMA was measured as the angle between the petrous ridge and the midline [9] (Figure 2). Cranial morphology was classified into three types according to two classification methods, respectively. In the CI method, brachycranic was defined as CI ≥ 80%, mesocranic as 75.0% < CI < 80%, and dolichocranic as CI ≤ 75%. In the PMA method, brachycranic was defined as PMA ≥ 54°, mesocranic as 46° < PMA < 54°, and dolichocranic as PMA ≤ 46° [1,9]. All measurements were conducted independently by two researchers, who identified the locations, lengths and angles. The measurements from each researcher were averaged.

### 2.4. Data Analysis

All data analyses for the measured variables were performed using SPSS statistical software (IBM SPSS Statistics ver. 23.0, IBM Co., Armonk, NY, USA). Comparisons of means by sex were analyzed using the independent samples *t*-test. Comparisons of means by age group were analyzed using the Kruskal–Wallis test, with post hoc analysis performed using Dunn’s test. Frequency analysis of cranial morphology classifications by sex and age group, as well as the relationship between CI and PMA, were conducted using Fisher’s exact test. Inter-class and Intra-class reliability were analyzed using values independently measured by two observers, and the classification criteria were based on Landis and Koch’s definition, with poor (0.00–0.21), fair (0.21–0.40), moderate (0.41–0.60), good (0.61–0.80), and excellent (0.81–1.00) [10]. After the authors prepared the draft of the manuscript, we used the generative AI tool ChatGPT (GPT-5, OpenAI) in a limited way to improve the English grammar and language. However, no AI support was used for the study design, data collection, analysis, or interpretation.

## 3. Results

### 3.1. Inter-Class Reliability Analysis

The two researchers conducted the measurements independently, identifying and performing the measurement points for MCL, MCW, and PMA. The inter-class reliability analysis showed that Cronbach’s α was 0.991 for MCL, 0.984 for MCW, and 0.739 for PMA. MCL and MCW were rated as excellent, while PMA was rated as good.

### 3.2. Comparison of Cranial Parameters by Sex

MCL and MCW were almost identical between males and females, showing no significant differences. CI was also comparable between sexes (85.4% vs. 85.9%), with mean values remaining within a narrow range. In contrast, PMA was significantly larger in males (55.5 ± 2.6°) than in females (54.5 ± 3.1°, *p* < 0.001), indicating a broader cranial angle in men (Table 1).

### 3.3. Comparison of Cranial Parameters by Age

**Figure 3:**
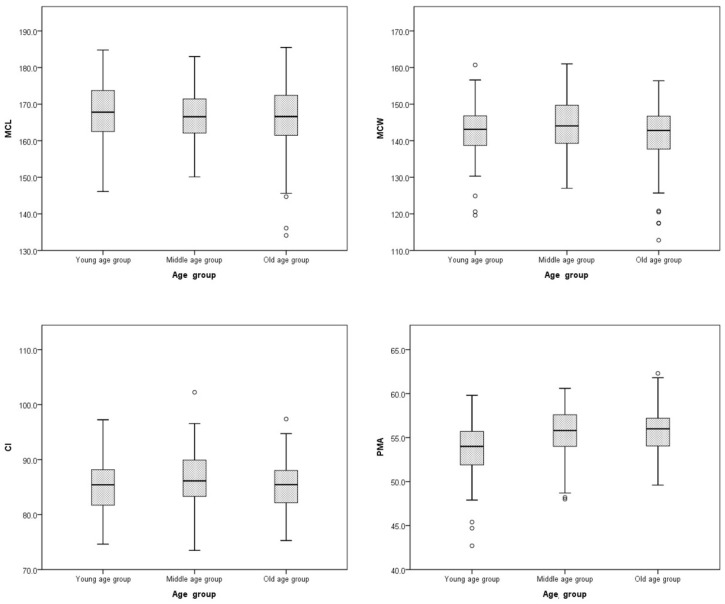
Boxplots of cranial parameter measurements by age group. CI, Cranial index; MCL, Maximum cranial length; MCW, maximum cranial width; PMA, Petrous ridge–midline angle.

MCL measured 167.9 ± 7.7 mm in the young age group, 166.8 ± 7.3 mm in the middle age group, and 166.0 ± 8.8 mm in the old age group, with no significant differences. MCW (143.0 ± 7.2, 144.1 ± 7.3, and 141.2 ± 7.3 mm, respectively) and CI (85.2 ± 4.5%, 86.5 ± 4.6%, and 85.1 ± 4.3%, respectively) also showed no significant variation across age groups, although the *p*-values were borderline. In contrast, PMA was significantly lower in the young age group (53.6 ± 3.1°) than in both the middle age group (55.5 ± 2.7°) and old age group (55.6 ± 2.6°, *p* < 0.001) (Figure 3, Table 2).

### 3.4. Frequency in Cranial Morphology According to CI by Sex and Age Group

Based on CI, most subjects were classified as brachycranic (89.4%), with smaller proportions of mesocranic (9.8%) and dolichocranic (0.8%). The brachycranic type was similarly predominant in both males (91.1%) and females (87.7%), showing no significant sex difference. Across age groups, the frequency of brachycranic remained high (86.0% in the young age group, 93.0% in the middle age group, and 87.8% in the old age group), without significant differences by age (Table 3).

### 3.5. Distribution of Cranial Morphology Based on PMA by Sex and Age Group

According to PMA, brachycranic accounted for 68.2% overall, followed by mesocranic (31.1%) and dolichocranic (0.8%). Brachycranic was significantly more frequent in males (73.7%) than in females (62.6%), while mesocranic was more common in females (35.8%) than in males (26.3%) (*p* = 0.022). By age, brachycranic increased markedly from 49.5% in the young age group to 74.6% in the middle age group and 74.8% in the old age group, indicating a significant age effect (*p* < 0.001) (Table 4).

### 3.6. Cross-Analysis of Cranial Morphology Classification According to CI and PMA

The cross-analysis of cranial classifications according to CI and PMA revealed that the most frequent cranial morphology was Brachycranic in both CI and PMA, with 228 out of 358 individuals. The second most frequent combination was Brachycranic in CI and Mesocranic in PMA, observed in 92 out of 358 individuals. Next, the frequency of individuals with Mesocranic in both CI and PMA, and those with Mesocranic in CI and Brachycranic in PMA, was equally 16 out of 358. Similarly, individuals with Dolichocranic in CI and Mesocranic in PMA, and those with Mesocranic in CI and Dolichocranic in PMA, both had a frequency of 3 out of 358. The frequency of cranial classifications based on CI and PMA showed a significant difference (*p* < 0.001) (Table 5).

## 4. Discussion

This study utilized PMCT images of deceased individuals obtained from the National Institute of Forensic Science to investigate the cranial morphology of Koreans. In previous studies on cranial morphology, analyses were conducted using skeletal remains of deceased individuals, living subjects, or plain X-ray images. More recently, cross-sectional images obtained in hospitals for diagnostic purposes have been widely utilized for cranial morphology research. However, CT images acquired in clinical settings require essential procedures such as obtaining consent for data usage and reviewing medical records, which makes securing large-scale datasets challenging. Therefore, this study considered PMCT images a suitable alternative, as they provide a large-scale dataset with a relatively balanced distribution of sex and age among Koreans.

In this study, the MCL of Koreans was 167.2 mm in males and 166.4 mm in females, while the MCW was 142.8 mm in both males and females. According to the literature, the MCL of Indians was 187.6 mm in males and 176.7 mm in females, and the MCW was 145.9 mm in males and 141.7 mm in females [11]. In Ghanaians, the MCL was 182.0 mm for males and 175.6 mm for females, and the MCW was 140.5 mm for males and 138.0 mm for females, with both sexes classified as Mesocranic [12]. These results indicate that while MCW values are relatively consistent across populations, the MCL of Koreans is comparatively shorter, resulting in a higher CI and the majority being classified as brachycranic.

In the cranial morphology, the brachycranic proportion among Koreans was 89.4%, which is similar to the findings of Choi et al. [13] (87.7%) and Hur et al. [14] (85.5%). In contrast, Iranians [15] showed the highest prevalence of dolichocranic at 65.7%, and Nigerians [16] showed 57.4% dolichocranic, while Turks [17] showed the highest prevalence of mesocranic at 42.5%, demonstrating distinct morphological differences among populations. These inter-population differences appear to be mainly influenced by the relative proportions of MCW and MCL. Koreans generally show greater MCW relative to MCL, producing a higher CI and a predominance of brachycranic, whereas Iranians and Nigerians tend to have longer MCLs with less transverse breadth, resulting in lower CI values. Turks, with intermediate proportions, mostly exhibit mesocranic. These results imply that such inter-population variation in CI reflects underlying developmental and adaptive patterns of the cranial vault rather than simple dimensional differences. Cranial morphology is also related to surgical approaches for cranial trauma, craniofacial reconstruction procedures, and spatial constraints in certain neurovascular conditions [18,19]. Therefore, understanding the morphological characteristics of Koreans can provide valuable information for surgical planning and pathological assessment.

In the cranial shape, the middle-aged group showed slightly higher CI values than the other groups. The difference was not statistically significant but indicated a borderline trend toward higher values (*p* = 0.050). Choi et al. [13] reported a similar trend, in which the CI increased with age and decreased after the age of 40, changes attributed to age-related alterations in MCL and MCW. For PMA, the values were 53.6° in the young age group, 55.5° in the middle age group, and 55.6° in the old age group, with the young age group showing significantly lower values than both the middle and old age groups (*p* < 0.001). These findings are consistent with Eskandary et al. [9], who observed an increase until approximately age 50, followed by stability into old age. While CI and PMA both vary with age, their patterns of change differ, and studies specifically addressing age-related variation in PMA remain limited. Understanding these patterns is important for anthropological age estimation in forensic cases, as well as for age-specific reference data in craniofacial developmental research. Moreover, the age-related differences in CI and PMA may suggest possible cranial remodeling associated with morphological maturation and degenerative changes, which should be further investigated in future studies.

In the CI classification, 91.1% of males and 87.7% of females were identified as brachycranic, and there was no significant sex difference (*p* = 0.533). This result suggests that cranial shape in Koreans is generally consistent between sexes, implying that sex has little effect on CI variation. Similar findings have been described in Thai populations, where 45.6% of males and 37.6% of females were classified as brachycranic [20]. In contrast, among Iranians, mesocranic was more frequent in females at 23.0% than in males at 6.6% [21], which indicates that sex-related differences in cranial morphology may vary across populations depending on genetic background and environmental factors.

By age group, the brachycranic proportion was 86.0% in the young age group, 93.0% in the middle age group, and 87.8% in the old age group, with no significant intergroup differences (*p* = 0.110). These results suggest that the predominance of brachycranic persists throughout adulthood without marked age-related changes. In contrast, an Iranian study by Eskandary et al. [9] showed a distinct age trend, with individuals under 15 years exhibiting more brachycranic, whereas those over 15 years had a higher frequency of dolichocranic. This indicates that while cranial morphology varies among populations, the degree to which age affects cranial shape may differ within each population.

In the PMA-based classification, 73.7% of males and 62.6% of females were brachycranic, a statistically significant sex difference (*p* = 0.022). By age group, the brachycranic proportion was 49.5% in the young age group, 74.6% in the middle age group, and 74.8% in the old age group, with significantly higher frequencies in the middle age and old age groups compared to the young age group (*p* < 0.001). These results demonstrate that while CI classification shows no variation by sex or age, PMA classification is more sensitive to detecting morphological differences across these demographic factors. Consequently, PMA may serve as a more effective parameter in forensic anthropology or archaeological contexts where sex-specific or age-specific cranial classification is required.

Previous studies have predominantly focused on classification methods using CI for cranial morphology, with limited research combining CI and PMA [4,8]. However, cranial morphology varies across populations, and using CI-based classification as the standard method and PMA-based classification as a complementary approach can provide more precise and detailed classification. In this study, a cross-analysis was performed to classify the complex cranial morphology using two classification methods, CI and PMA. As a result, the most frequent cranial morphology was brachycranic for both CI and PMA, accounting for 228 out of 358 subjects (71.3%). The second most frequent type was brachycranic for CI and mesocranic for PMA, accounting for 92 out of 358 subjects (28.7%). In contrast, a study on Iranians [9] showed that the highest frequency in the cross-analysis of CI and PMA was dolichocranic, with a frequency of 32.8% overall, followed by mesocranic for CI and dolichocranic for PMA (22.4%). Compared to our study, where the majority of subjects were brachycranic and mesocranic, the majority of subjects in Iran were dolichocranic and mesocranic. Thus, utilizing both CI and PMA in combination could serve as valuable data for more diverse and detailed cranial classification in racial comparisons. Additionally, more expanded studies would be essential to compare these results by population and classification. These differences between CI- and PMA-based classifications may reflect the complex nature of cranial shape, as linear measurements and angular parameters represent different aspects of cranial structure. This suggests that cranial morphology cannot be fully explained by a single index and that a combined approach using both CI and PMA provides a more realistic understanding of cranial variation.

When compared with cadaver-based studies, the CI and PMA values obtained in this study were about 3% and 8% higher, respectively [22]. This difference does not necessarily mean that either method is inaccurate. In cadaveric measurement, even though the skull is measured directly in three dimensions, the observer’s visual angle and the difficulty of defining consistent reference planes on curved surfaces can still affect accuracy. In contrast, 2D PMCT imaging may involve projection-related distortion when representing three-dimensional structures. Taken together, these variations are more likely due to methodological differences rather than real anatomical disparities. It would be valuable to develop improved measurement techniques that can reduce such methodological discrepancies between imaging- and cadaver-based analyses.

Nevertheless, our study has several limitations. First, since this study conducted measurements based on 2D PMCT images, errors may occur depending on the shooting angle of the corpse. Additional research, such as measurements using 3D reconstruction or dissections of cadavers for more accurate measurements, is needed. Second, errors may occur in angle measurements using CT images, and additional research will be needed to secure a measurement method in a 3D environment with relatively high reproducibility. Third, there is a possibility that a type 1 error may occur because correction for multiple comparisons was not applied.

## 5. Conclusions

In this study, CI and PMA methods were used to classify the cranial morphology of Koreans using PMCT images. In the classification of skull shape using both CI and PMA methods, the brachycranic type showed the highest frequency. In CI methods there were no significant differences in frequency by sex or age (*p* = 0.533, *p* = 0.110). However, in PMA methods the frequency of the brachycranic type in men was significantly higher than in women (*p* = 0.022) and there was a significant difference in the frequency of cranial morphology by age (*p* < 0.001). The results of cranial morphology classification targeting Koreans are expected to provide useful basic data for clinical and forensic use.

## Figures and Tables

**Table 1:** Comparison of mean values of cranial parameters by sex.

Parameter	Male (*n* = 179)	Female (*n* = 179)	Total (*n* = 358)	*p*
MCL (mm)	167.2 ± 8.9	166.4 ± 6.9	166.8 ± 8.0	0.332
MCW (mm)	142.8 ± 8.7	142.8 ± 6.2	142.8 ± 7.5	0.996
CI (%)	85.4 ± 4.2	85.9 ± 4.8	85.7 ± 4.5	0.304
PMA (°)	55.5 ± 2.6	54.5 ± 3.1	55.0 ± 2.9	0.001

The data are expressed by mean ± SD. CI, Cranial index; MCL, Maximum cranial length; MCW, maximum cranial width; PMA, Petrous ridge–midline angle.

**Table 2:** Comparison of mean values of cranial parameters by age group.

Parameter	Young Age		Middle Age		Old Age		*p*
	Mean ± SD	Median (Q1, Q3)	Mean ± SD	Median (Q1, Q3)	Mean ± SD	Median (Q1, Q3)	
MCL (mm)	167.9 ± 7.7	167.8 (162.4, 178.0)	166.8 ± 7.3	166.5 (162.0, 171.4)	166.0 ± 8.8	166.6 (161.4, 172.4)	0.322
MCW (mm)	143.0 ± 7.2	143.1 (138.6, 147.0)	144.1 ± 7.3	144.0 (139.3, 149.7)	141.2 ± 7.3	142.8 (137.6, 146.9)	0.058
CI (%)	85.2 ± 4.5	85.4 (81.6, 88.2)	86.5 ± 4.6	86.1 (83.2, 89.9)	85.1 ± 4.3	85.4 (82.0, 88.0)	0.050
PMA (°)	53.6 ± 3.1	54.0 (51.8, 55.7)	55.5 ± 2.7	55.8 (53.9, 57.6)	55.6 ± 2.6	56.0 (54.0, 57.2)	<0.001

The data are expressed by mean ± SD. The comparison of means was performed using the Kruskal–Wallis test, with Dunn’s test applied for post hoc analysis. SD, standard deviation; Q1, first quartile; Q3, third quartile; CI, Cranial index; MCL, Maximum cranial length; MCW, maximum cranial width; PMA, Petrous ridge–midline angle.

**Table 3:** Frequency in cranial morphology according to CI by sex and age group.

CI	Sex			*p*	Age Group				*p*
	Male	Female	Total		Young Age	Middle Age	Old Age	Total	
Brachycranic (CI ≥ 80%)	163 (91.1)	157 (87.7)	320 (89.4)	0.533	80 (86.0)	132 (93.0)	108 (87.8)	320 (89.4)	0.110
Mesocranic (75% < CI < 80%)	15 (8.4)	20 (11.2)	35 (9.8)		12 (12.9)	8 (5.6)	15 (12.2)	35 (9.8)	
Dolichocranic (CI ≤ 75%)	1 (0.6)	2 (1.1)	3 (0.8)		1 (1.1)	2 (1.4)	0 (0.0)	3 (0.8)	
Total	179 (100.0)	179 (100.0)	358 (100.0)		93 (100.0)	142 (100.0)	123 (100.0)	358 (100.0)	

The data are presented as number (percent). CI, Cranial index.

**Table 4:** Frequency in cranial morphology according to PMA by sex and age group.

PMA	Sex			*p*	Age Group				*p*
	Male	Female	Total		Young Age	Middle Age	Old Age	Total	
Brachycranic (PMA ≥ 54˚)	132 (73.7)	112 (62.6)	244 (68.2)	0.022	46 (49.5)	106 (74.6)	92 (74.8)	244 (68.2)	<0.001
Mesocranic (46˚ < PMA < 54˚)	47 (26.3)	64 (35.8)	111 (31.1)		44 (47.3)	36 (25.4)	31 (25.2)	111 (31.1)	
Dolichocranic (PMA ≤ 46˚)	0 (0.0)	3 (1.7)	3 (0.8)		3 (3.2)	0 (0.0)	0 (0.0)	3 (0.8)	
Total	179 (100.0)	179 (100.0)	358 (100.0)		93 (100.0)	142 (100.0)	123 (100.0)	358 (100.0)	

The data are presented as number (percent). PMA, Petrous ridge–midline angle.

**Table 5:** Cross-analysis of cranial morphology classification according to CI and PMA.

CI		PMA			Total	*p*
		Brachycranic (PMA ≥ 54˚)	Mesocranic (46˚ < PMA < 54˚)	Dolichocranic (PMA ≤ 46˚)		
Brachycranic (CI ≥ 80%)	Count	228	92	0	320	<0.001
	% within rows	71.3%	28.7%	0.0%	100.0%	
	% within columns	93.4%	82.9%	0.0%	89.4%	
Mesocranic (75% < CI < 80%)	Count	16	16	3	35	
	% within rows	45.7%	45.7%	8.6%	100.0%	
	% within columns	6.6%	14.4%	100.0%	9.8%	
Dolichocranic (PMA ≤ 75%)	Count	0	3	0	3	
	% within rows	0.0%	100.0%	0.0%	100.0%	
	% within columns	0.0%	2.7%	0.0%	0.8%	
Total	Count	244	111	3	358	
	% within rows	68.2%	31.0%	0.8%	100.0%	
	% within columns	100.0%	100.0%	100.0%	100.0%	

The data are presented as number (percent). CI, Cranial index; PMA, Petrous ridge–midline angle.

## Data Availability

The datasets used and/or analyzed during the current study are available from the corresponding author upon reasonable request.
